# The Current Landscape of Marijuana and Pharmacogenetics

**DOI:** 10.7759/cureus.1525

**Published:** 2017-07-30

**Authors:** Kerry Anne Rambaran, Michael Chu, Tyler B Johnson, Saeed K Alzghari

**Affiliations:** 1 Department of Clinical Sciences, Keck Graduate Institute; 2 Gulfstream Genomics, Gulfstream Diagnostics

**Keywords:** marijuana, medical marijuana, genetics, pharmacogenetics, cannabis, marijuana legalization

## Abstract

The treatment of medical conditions with cannabis and cannabinoid compounds is advancing. Although there are numerous reports related to the genetic variations of the cannabinoid receptor, a lack of studies that examine the relationship between other pharmacogenetic markers and health outcomes currently exists. Herein, we advocate for the legalization of marijuana in the United States in order to perform more randomized controlled trials to help elucidate the role of other pharmacogenetic targets and cannabis for use in clinical practice.

## Editorial

In 1937, the United States (US) passed a federal law banning cannabis. Thereafter, in 1976 through the Investigational New Drug compassionate access program, qualified individuals could receive no more than nine pounds of cannabis from the federal government. Two decades later, in 1996, California became the first state to legalize medical marijuana, although it remains classified as illegal from the federal standpoint. To date, there are 29 states with recognized medical marijuana programs and 17 other states have approved low delta-9-tetrahydrocannabinol (THC), high cannabidiol (CBD) for limited medical purposes.

Currently, the two main cannabinoids derived from the marijuana plant utilized in studies are CBD and THC. Both are beneficial in controlling pain and inflammation. CBD has shown some benefit in epilepsy in preliminary clinical trials. CBD is available as Epidiolex^®^ and current trials focus on severe, early-onset, orphan, treatment-resistant syndromes including Dravet syndrome, Lennox-Gastaut syndrome, tuberous sclerosis complex and infantile spasms. Additionally, nabiximol (Sativex^®^), which contains both CBD and THC, received approval for muscle spasticity due to multiple sclerosis in over 29 countries outside the US. However, the Food and Drug Administration (FDA) is yet to approve the use of any of these agents. To that end, the FDA has approved two prescription drugs, dronabinol (Marinol^®^) and nabilone (Cesamet^®^), based on a component of marijuana that is used in nausea and vomiting secondary to chemotherapy.

Given that the two main cannabinoids may aid in pain and inflammation control, some articles postulate there may be a decrease in opioid use in individuals that use cannabinoid derivatives [1-2]. Moreover, Bradford and colleagues reported, from 2010 to 2013, Medicare Part D individuals saw a significant reduction in the use of prescription drugs when the implementation of a marijuana law occurred with an estimated savings of $165.2 million in 2013 [3]. Furthermore, Powell, et al. reported that legally protected medical marijuana dispensaries, in addition to marijuana laws, were associated with a decrease in opioid prescribing, treatment admission for opioid abuse, self-reporting of non-prescription opioid use and deaths resulting from prescription opioid overdose [4]. However, these studies suggest more of an associative relationship rather than a causative one. Thus, there is a need for more studies. As such, the National Institute on Drug Abuse is currently executing several trials that look at the effect of access to medical marijuana for substance use, longitudinal trajectories in marijuana use, pain and functioning, and the impact of medical marijuana policies on health outcomes.

The literature associated with the pharmacogenetics of cannabinoids includes receptor genes, transport and action genes, metabolism genes, endocannabinoid biosynthesis and bioactivation genes. However, the most common gene found across studies is the cannabinoid receptor 1 gene as it is the best understood [5]. Nonetheless, inconsistency in results across studies that look at pharmacogenetic variations or cannabinoids exists. In order to utilize cannabinoids as a treatment agent, we must first ascertain the pharmacogenetic markers in various populations. While it is beneficial to conduct clinical trials in target disease populations, we must consider robust clinical trials related to the pharmacogenetics of cannabinoids.

We searched for completed and ongoing studies via ClinicalTrials.gov using the terms “marijuana”, “cannabis”, “genetics”, and “pharmacogenetics” that resulted with four relevant trials (Table 1). The sample size of the four trials ranges from 60 to 162 patients with three utilizing THC versus placebo. Of note, three of the trials are investigating the catechol-o-methyltransferase gene polymorphism with current research efforts focused on its role in psychiatry. Additionally, of the four trials, one is ongoing, one was completed and the status of two is unknown and no results or outcomes are currently available.

**Table 1:** Current pharmacogenetic trials involving marijuana. CB1: Cannabinoid receptor 1; COMT: Catechol-o-methyltransferase; GABA: Gamma-aminobutyric acid; PET: Positron emission tomography; THC: Tetrahydrocannabinol.

Trial	Trial status	Study title	Gene polymorphisms investigated	Sample size	Intervention	Comparator	Primary outcome	Secondary outcome
NCT00678730	This study is ongoing, but not recruiting participants	Pharmacogenetics of cannabinoid response	COMT	162	Drug: delta-9-tetrahydrocannabinol	Active comparator: Very low dose (0.005 mg/kg = 0.35 mg in a 70 kg individual) THC, dissolved in ethanol. Low dose (0.025 mg/kg = 1.75 mg in a 70 kg individual) THC, dissolved in ethanol. Medium dose (0.05 mg/kg = 3.5 mg in a 70 kg individual) THC, dissolved in ethanol. Placebo comparator: small amount of ethanol (quarter teaspoon)	This study is ongoing	N/A
NCT01565174	Unknown	The pharmacogenetic and brain mechanisms associated with cannabis-induced psychosis	Dopamine, GABA, glutamate, and cannabis receptor CB1	100	N/A	N/A	N/A	N/A
NCT02492074	Unknown	Gene-environment-interaction: influence of the COMT genotype on the effects of different cannabinoids - a PET study	COMT	60	Drug: delta-9-tetrahydrocannabinol Drug: cannabidiol Drug: placebo	Placebo comparator: placebo subjects receive corresponding delta-9-tetrahydrocannabinol and cannabidiol placebo capsules	N/A	N/A
NCT02487381	Completed	Gene-environment-interaction: influence of the COMT genotype on the effects of different cannabinoids	COMT	60	Drug: delta-9-tetrahydrocannabinol Drug: cannabidiol Drug: placebo	Placebo comparator: placebo subjects receive corresponding delta-9-tetrahydrocannabinol and cannabidiol placebo capsules	Not posted	Not posted

Clearly, there is a dearth of clinical trials relating to pharmacogenetics and cannabis. In order to ascertain if these derivatives or compounds are safe and efficacious in various genetic polymorphisms and patient populations, we need to engage in more robust clinical trials involving genetic testing. However, the legal constraints against marijuana in the US limit progress. Currently, there is only one source of marijuana for research in the US through the University of Mississippi. Research utilizing cannabis at-large is severely limited and a call for legalization at the federal level to remove its Drug Enforcement Agency schedule I status would alleviate this difficulty. The political stigma associated with marijuana needs to be set aside and US lawmakers need to take a serious look at the benefits of cannabis for the good of society as a whole.
